# How do Effort, Reward, and Their Combined Effects Predict Burnout, Self-rated Health, and Work-family Conflict Among Permanent and Fixed-term Faculty?

**DOI:** 10.1093/annweh/wxac094

**Published:** 2023-01-06

**Authors:** Anna Sofia Tanimoto, Anne Richter, Petra Lindfors

**Affiliations:** Department of Psychology, Stockholm University, 106 91, Stockholm, Sweden; Department of Psychology, Stockholm University, 106 91, Stockholm, Sweden; Department of Learning, Informatics, Management and Ethics, Karolinska Institutet, 171 77, Stockholm, Sweden; Department of Psychology, Stockholm University, 106 91, Stockholm, Sweden

**Keywords:** HEI, psychosocial work environment, structural equation modeling, temporary employment, work-home interference

## Abstract

Employment conditions and psychosocial factors have been linked to various health-related outcomes in different occupational groups, but few studies focus on the conditions in academia. This study explores the effects of effort, reward, and their interaction to explain health-related outcomes, namely burnout, self-rated health, and work-family conflict among academic faculty in Sweden. We also explore these effects among those with permanent and fixed-term employment contracts. Questionnaire data, collected online in 2016, came from 2335 employees (57% women) with a doctoral degree, working at a Swedish higher education institution. Latent moderation analysis combined with multi-group analysis was conducted. Main effects of effort were found for all health-related outcomes revealing that effort was associated with higher burnout, poorer self-rated health, and greater work-family conflict. Reward was negatively associated with burnout and self-rated health revealing that reward reduced burnout and improved self-rated health. The interaction between effort and reward was significantly associated with all outcomes among permanent contract employees, but was non-significant among those with fixed-term contracts. This may suggest that fixed-term faculty are less affected by the presence or lack of reward. Overall, the findings emphasize the importance of the psychosocial work environment to understand health-related consequences for permanent and fixed-term faculty with a doctoral degree.

**What’s Important About This Paper**

This study furthers our understanding of the psychosocial work environment and employment conditions among academic faculty in Sweden. The findings reveal that although reward moderated the relationship between effort and the health-related outcomes among permanent faculty, the same relationship was not apparent among fixed-term faculty. As various types of non-permanent employment contracts exist in the academic context, it is important to better understand how academic faculty with different contractual statuses experience their working conditions in relation to various health-related outcomes.

## Introduction

Recently, the work environment in academia has come under scrutiny with accumulated findings depicting the ubiquity of stressful working conditions (Urbina-Garcia, 2020). As temporary employment contracts have become increasingly commonplace (OECD, 2021), so have concerns about their influence on the psychosocial work environment (Urbina-Garcia, 2020). Studies of psychosocial factors and employee outcomes among faculty in academia suggest that the current conditions may contribute to poor health (Zábrodská *et al.*, 2018; Kinman, 2019; Urbina-Garcia, 2020) and work-home interference (Zábrodská *et al.*, 2018). However, few empirical studies have examined the psychosocial working conditions in the Swedish context and their implications for individuals working at higher education institutions. Moreover, given the prevalence of temporary contracts in academia (around 30% in Sweden; Swedish Higher Education Authority, 2020), few studies have investigated the role of employment contracts in the relationship between the psychosocial work environment and health-related outcomes. Research has shown differences between permanent and temporary contract employees regarding how psychosocial factors are associated with health and well-being (De Cuyper *et al.*, 2010; Inoue *et al.*, 2010; Van den Tooren and De Jong, 2014), but their generalization to academia remains unclear. Hence, the present study sets out to: (i) investigate the relationship between the psychosocial working conditions and health-related outcomes (i.e. burnout, self-rated health, and work-family conflict) among academic faculty in Sweden, and (ii) empirically contribute to the research on psychosocial working conditions, contract type, and associated health-related outcomes in academia.

### Effort-reward imbalance

The effort-reward imbalance (ERI) model is one of the most prevalent occupational health models to explain work-related stress and adverse health-related employee outcomes including burnout, self-rated health, and work-family conflict (Van Vegchel *et al.*, 2005a; Siegrist and Li, 2016; Gorgievski *et al.*, 2019). ERI accounts for various demanding aspects of work which require employee effort, in addition to compensation for said effort, namely rewards (Siegrist, 1996). Rewards are assumed to counterbalance effort and consist of three components: (i) esteem, (ii) job security, and (iii) promotion prospects. Stemming from social reciprocity norms, employees expect to be rewarded to an extent befitting the effort they expend on their work, and imbalance is said to occur when these expectations of reciprocity are not met, for instance when an employee exerts high effort, but perceives low reward. This perceived imbalance can trigger negative stress reactions, which if sustained, put individuals at risk for adverse health (Siegrist, 1996). Thus, ERI can be used to gauge employee perceptions of the psychosocial work environment. Although the model was originally used to investigate cardiovascular outcomes (Siegrist, 1996), its utility to predict various health-related outcomes is well-documented (Van Vegchel *et al.*, 2005a; Siegrist and Li, 2016).

### Burnout and overall health

Burnout (BO), a stress-related syndrome (Maslach *et al.*, 2001), is a common consequence for employees with prolonged exposure to stressful psychosocial working conditions (Canu *et al.*, 2021). After an extended period of time, characterized by efforts to meet increasing or high work demands and insufficient rewards, individuals’ resources to handle these conditions may erode, potentially increasing vulnerability to BO. Indeed, research supports the relationship between ERI and BO (Hasselhorn *et al.*, 2004) and specifically, ERI and exhaustion (Aronsson *et al.*, 2017; Leineweber *et al.*, 2021), the first and most central BO component (Maslach *et al.*, 2001).

Apart from BO, ERI has been associated with adverse health outcomes, including physical health (Van Vegchel *et al.*, 2005a), mental health (Stansfeld and Candy, 2006), and self-rated health (SRH; Niedhammer *et al.*, 2004; Siegrist *et al.*, 2009; Leineweber *et al.*, 2010). Yet, it is unknown whether these findings generalize to academia. One European study of academics investigated ERI in relation to self-reported physical and mental health outcomes (Kinman, 2019). Here, however, reward components were investigated separately which did not account for any aggregated effects of reward for these outcomes. Thus, research investigating the relationships between psychosocial factors and faculty health is needed.

### Effort-reward imbalance and work-family conflict

Besides predicting health outcomes, ERI is relevant for other health-related outcomes associated with stressful working conditions, including conflict between work and family domains. Interference between these domains, referred to as work-to-family conflict (WFC) and family-to-work conflict (FWC), involves an inter-role conflict where role responsibilities in one domain conflict with those in the other (Greenhaus and Beutell, 1985). Given the centrality of the work domain in this study of faculty, this research focuses solely on WFC.

Strain and time-based conflict are mechanisms through which WFC can occur (Greenhaus and Beutell, 1985; Voydanoff, 2004). For instance, strain-based work demands, including a heavy workload, may elicit negative reactions, carrying over to the family domain. Time-based demands, such as working overtime, may limit time for other tasks including family responsibilities. Both mechanisms might explain why conflict occurs. Considering that effort refers to work demands, including time pressure and interruptions (Siegrist, 1996), these are comparable to the strain and time-based demands identified as WFC antecedents (Greenhaus and Beutell, 1985; Voydanoff, 2004; Byron, 2005). For example, positive associations have been found between work stressors and strain-based conflict, specifically WFC (Voydanoff, 2004; Byron, 2005). While effort might contribute to strain or time-based conflict, reward may bring about positive emotions, and contribute positive spillover in the family domain, thereby reducing WFC. An imbalance between effort and reward may elicit negative emotions causing irritation and add negative spillover, hindering individuals from fulfilling family responsibilities.

Few studies have explored ERI in relation to WFC (Kinman and Jones, 2008; Gorgievski *et al.*, 2019; Jerg-Bretzke *et al.*, 2020) and results vary. While Jerg-Bretzke and colleagues (2020) find no association between ERI and WFC, some studies only report significant effects of effort on WFC (Gorgievski *et al.*, 2019). Other findings from academic employees show an interaction between effort and reward for WFC (Kinman and Jones, 2008). In these studies, effort explained more variance in WFC than reward, but further research is needed to clarify the mechanisms, particularly among faculty.

### Effort, reward and their interaction

The ERI literature has mainly investigated the predictive capacity of *imbalance*, operationalized using a ratio (Van Vegchel *et al.*, 2005a; Leineweber *et al.*, 2021). Yet an often-overlooked model assumption is that imbalance has predictive capacity over and above the independent effects of effort and reward. Examining the individual contributions of effort and reward are important for understanding the effects of demanding aspects of work, and the various resources that can impact health-related outcomes. Some research reported only main effects of effort predicting exhaustion and WFC, while lack of reward only predicted exhaustion (Gorgievski *et al.*, 2019), and low reward was associated with exhaustion (Aronsson *et al.*, 2017). These empirical inconsistencies may challenge theoretical assumptions of effort and reward.

Besides investigating main effects of effort and reward, a review of ERI studies recommends analyzing continuous variables, making use of all available information, rather than collapsing data and using cut-offs (Van Vegchel *et al.*, 2005a). Consequently, the co-occurrence of effort and reward has since been examined using alternative methods to the ratio, including the multiplicative approach (Van Vegchel *et al.*, 2005b). This introduces a multiplicative term, an *interaction* between effort and reward, testing whether reward operates as a moderator, strengthening or weakening the effects of effort on the outcome (Van Vegchel *et al.*, 2005b). This interaction implies that when effort and reward are high, reward acts as a buffer, whereas when effort is high and reward is low, reward has an exacerbating effect. Where the ERI ratio only indicates the presence or extent of *imbalance,* the interaction accounts for the full range of possible combinations of effort and reward, to explain outcomes in relation to the combination and intensity of effort and reward. Thus, this approach focuses less on the specifics of *imbalance* per se, and extends the possible ways in which effort and reward may relate to strain. Existing studies report significant interaction effects related to sickness absence (Van Vegchel *et al.*, 2005b) and WFC (Kinman and Jones, 2008), but further investigation is warranted.

### The role of contract type

There are various employment types in academia, each with unique implications for the individual employees and for the psychosocial working conditions. While some argue that permanent, versus fixed-term employment, is associated with better psychosocial working conditions (Aronsson *et al.*, 2002), the situation in academia in Sweden may be more complex. Largely, the implications of psychosocial working conditions and permanent versus fixed-term employment for faculty are understudied.

Comparisons of permanent and temporary employees in other sectors are inconclusive, partly due to the heterogeneity of employment types which often fall into a category of *temporary employment*, for instance fixed-term, but also on-call employments. Comparing permanent and various types of temporary contracts, one study found adverse health effects for permanent, but not on-call employees. Moreover, no differences emerged between permanent and fixed-term employees (Bernhard-Oettel *et al.*, 2005). Other findings, where the research only distinguished between permanent and temporary contracts, show similar health effects for permanent as opposed to temporary employees (De Cuyper *et al.*, 2010; Van den Tooren and De Jong, 2014). Similar research investigating diverse employment types in relation to health show no differences between permanent and fixed-term contract employees (Virtanen *et al.*, 2003). This may be attributed to the relative stability of these employment types. Differences between permanent and fixed-term employees may also decrease when fixed-term contracts have an extended duration and employment benefits and conditions more closely resemble those of permanent employees (De Cuyper *et al.*, 2010). Given the uniqueness of employment conditions in academia, it is important to consider the conditions characterizing permanent and fixed-term contracts.

Some research has investigated differences between permanent and temporary employees in academia, focusing on employee well-being, and job satisfaction (Fontinha *et al.*, 2018; Castellacci and Viñas-Bardolet, 2021). Among faculty in Europe, permanent contracts were positively associated with job satisfaction. Specifically, permanent contracts were more important for job satisfaction among those mid-career (Castellacci and Viñas-Bardolet, 2021). Other findings from the UK revealed that the absence of work stress was associated with well-being among both permanent and fixed-term employees. However, the relationship was weaker for temporary employees (Fontinha *et al.*, 2018). Conditions in academia clearly require investigation, especially in the Swedish context. Not only are findings from other sectors varied and unapplicable, but the paucity of research regarding contract type in academia means that it is important to explore how contract type is associated with psychosocial working conditions and health-related outcomes.

### Aims

The overall aim of this study is to examine effort, reward, and their interaction in relation to BO, SRH, and WFC among faculty with permanent and fixed-term employment contracts, respectively. Consequently, this study tests the following hypotheses:

*Hypothesis 1a–c*: Effort is positively related to (a) BO, (b) SRH, and (c) WFC.
*Hypothesis 2a–c*: Reward is negatively related to (a) BO, (b) SRH, and (c) WFC.
*Hypothesis 3a–c*: Reward moderates the relationship between effort and health-related outcomes including (a) BO, (b) SRH, and (c) WFC.

Moreover, taking an exploratory approach, we investigate how these relationships play out among faculty with permanent and fixed-term contracts, respectively.

## Materials and methods

### Data collection and sample

In 2016, 18 687 members of a union for teachers and researchers in academia in Sweden were invited to an online questionnaire study, to be completed in Swedish or English. Participants were informed of the study and asked to provide informed consent. Twenty-five percent (*n* = 4780) responded whereof 4762 individuals provided valid informed consent. This study included those in possession of a doctoral degree, working at a Swedish higher education institution, who provided key demographic details, resulting in an effective sample-size of 2335 faculty (average age: 48 years; 57% women; permanent contracts: 78%). The Stockholm Regional Ethics Committee (2016/694-31) approved the research.

### Measures

#### Effort

Effort was measured with three items (example item: *There are many interruptions and disturbances in my job*; Siegrist *et al.*, 2009; Leineweber *et al.*, 2010). Ratings were made on a 5-point scale (1: strongly disagree; 5: strongly agree; *α* = 0.77).

#### Reward

Seven items measured reward, which includes three dimensions: esteem, security, and promotion (example item: *Considering all my efforts and achievements, my work prospects are adequate*; Siegrist *et al.*, 2009; Leineweber *et al.*, 2010). Ratings were made on a 5-point scale (1: strongly disagree; 5: strongly agree; *α* = 0.80).

#### Burnout

Nine adjectives from the Scale of Work Engagement and Burnout (SWEBO) measured state mood of burnout in the past 2 weeks (Hultell and Gustavsson, 2010). Burnout contains three dimensions: exhaustion, disengagement, and inattentiveness. Ratings were made on a 4-point scale (1: not at all; 4: all the time; *α* = 0.86) where high scores indicate higher burnout.

#### Self-rated health

Considered a holistic indicator of health status (Idler and Benyamini, 1997), a single-item measure of health was used, asking respondents to rate their overall health. Ratings were made on a 5-point scale (1: very good; 5: very poor).

#### Work-family conflict

Work-family conflict was measured with two items (example item: *How often does it happen that your work or career interferes negatively with your private life?*; Frone *et al.*, 1992). Ratings were made on a 5-point scale (1: never; 5: always; *α* = 0.82).

#### Contract type and covariates

Contract type was divided into two categories: permanent and fixed-term. Fixed-term included, for instance, substitutes, post docs, and probationary contracts or stipends.

Covariates included age, gender (0 = female; 1 = male), civil status, (0 = single; 1 = partnered), and children aged 12 and younger at home (0 = no; 1 = yes). Previous research of faculty shows that health-related problems are more prevalent among women, especially younger women (Lohela-Karlsson *et al.*, 2018). Also, age and gender have been related to burnout in academic staff (Zábrodská *et al.*, 2018), while children and civil status have been related to work-family interference (Byron, 2005).

### Data preparation and analytic strategy

To ensure normal distribution of the data, skewness and kurtosis were screened. Skewness was not greater than three, and kurtosis no larger than ten (Weston and Gore, 2006; Kline, 2016). None of the analysis variables were highly correlated (*r* > 0.85; Weston and Gore, 2006), suggesting no multicollinearity. Analyses to identify mechanisms of missing data revealed that BO items were not missing completely at random; (*χ*^2^ = 150.49, *df* = 104, *P* < 0.05). The full information maximum likelihood estimation method for missing data was implemented in the structural equation models in Mplus Version 8.6. Data screening and production of the correlation table was conducted in SPSS Version 26.

First, a measurement model was specified in order to assess the precision of measurement and internal factor structure across all latent variables under investigation. Reward and BO were modeled as second-order factors, predicting the first-order factors (intercepts fixed to zero for model identification), esteem, security, promotion, and exhaustion, disengagement, inattentiveness, respectively. Effort and WFC were modeled as first-order factors and SRH as an observed variable. The standardized factor loadings for all indicators were statistically significant, *P* < 0.001, ranging from 0.45 to 0.92. Based on recommendations, indices to evaluate adequate model fit included CFI > 0.90, TLI > 0.90, SRMR < 0.08, and RMSEA < 0.08 (Hu and Bentler, 1999). The measurement model showed acceptable fit (CFI = 0.93; TLI = 0.92; SRMR = 0.05; RMSEA = 0.06), thus allowing for continued analyses and hypothesis testing in the structural models.

Next, three structural models were estimated using the maximum likelihood robust estimator: Model 1, Model 2, and Model 3. In all three models, contract type was stratified. The hypotheses and exploratory research question were tested in Model 3. In Model 1, multiple groups were specified by contract type (permanent and fixed-term) using the GROUPING command in Mplus. Then, the covariates (i.e. age, gender, civil status, and children), the dependent variables BO, SRH, and WFC were regressed on the latent factors of effort and reward. This model was specified as a baseline structural model in order to assess model fit (using traditional model fit statistics). An extension of the first model, Model 2, only differed from Model 1 in that it was specified as a mixture model, and the KNOWNCLASS command was used to account for the two groups. This mixture model produces no traditional fit statistics, but acts as an approximation of the previous model, and is necessary for model comparison when conducting a log likelihood difference test. The final model, Model 3, simply introduced a latent interaction term to the previous model, estimating a latent moderation model (LMS) across the multiple groups. Compared with partially latent and non-latent models, the LMS approach provides greater precision in estimating interaction effects as measurement error is taken into account during estimation (Klein and Moosbrugger, 2000). Using the Mplus XWITH function, the interaction between effort and reward was specified and included as a predictor.

As traditional goodness of fit indices (e.g. CFI and SRMR) cannot be obtained when estimating mixture models, the mixture models (Models 2 and 3) were compared for fit using the Akaike information criterion (AIC), Bayesian information criterion (BIC), and Sample-size adjusted Bayesian information criterion (SABIC), where lower values indicate better fit (Kline, 2016). Model selection was also based on the log likelihood difference test comparing Models 3 and 2, where a significant difference indicates that the model without the interaction represents a significant loss of fit (Satorra and Bentler, 2010).

Interactions were plotted using R-package rhdf5. Simple slopes analyses were conducted to provide information regarding the strength of the effect at −1, 0, and +1 standard deviations of the moderator, reward.

## Results

### Descriptive statistics and correlations

Table 1 presents descriptive statistics and correlations.

**Table 1.** Means, standard deviations, and Pearson correlations, for study variables (complete cases). Correlations above the diagonal represent fixed-term contracts (*n* = 455) and those below represent permanent contracts (*n* = 1596).

Variable	Permanent	Fixed	1.	2.	3.	4.	5.	6.	7.	8.	9.
	*M* (SD)	*M* (SD)									
1. Age	50 (8.45)	40 (6.88)	–	−.10^*^	.03	−.11^*^	.07	−.14^**^	−.03	.09	.04
2. Gender (male)	44%	40%	.03	–	.02	.04	−.05	.05	−.04	−.11^*^	−.07
3. Civil status (partnered)	85%	83%	−.07^**^	.13^**^	–	.35^**^	.12^**^	−.11^*^	−.03	−.10^*^	−.03
4. Children (under 12)	37%	56%	−.57^**^	.05^*^	.18^**^	–	.10^*^	−.08	.06	.04	.06
5. Effort	3.97 (.78)	3.76 (.83)	−.03	−.13^**^	.00	.00	–	−.29^**^	.32^**^	.23^**^	.50^**^
6. Reward	3.16 (.78)	2.70 (.79)	−.00	.02	.07^**^	.02	−.25^**^	–	−.40^**^	−.31^**^	−.27^**^
7. BO	1.77 (.50)	1.92 (.50)	−.19^**^	−.09^**^	−.09^**^	.10^**^	.34^**^	−.44^**^	–	.51^**^	.34^**^
8. SRH	2.17 (.91)	2.27 (.89)	−.05	−.07^**^	−.11^**^	.00	.24^**^	−.30^**^	.50^**^	–	.27^**^
9. WFC	3.55 (.86)	3.60 (.83)	.00	−.11^**^	−.02	−.00	.53^**^	−.24^**^	.33^**^	.27^**^	–

*n* = 2051.

–, Not applicable; Fixed, fixed-term; BO, burnout; SRH, self-rated health; WFC, work-family conflict.

^**^ *P* < 0.01,

^*^ *P* < 0.05, two-tailed.

### Model fit and model comparison

Table 2 presents model fit indices. Model 1 demonstrated adequate fit. As for Models 2 and 3: although a decreasing AIC supported selecting Model 3, BIC was largest in Model 3. However, SABIC, which adjusts for the penalty imposed on BIC for large sample sizes, supported selecting Model 3. Furthermore, the loglikelihood difference, testing whether Model 2 represented a significant loss in fit to Model 3, was significant, *D =* 30.98, *P* < 0.05, indicating that the interaction model better approximated the data.

**Table 2.** Summary of model fit indices for the three structural equation models and log likelihood difference test for model comparison.

Model	CFI	TLI	SRMR	RMSEA	AIC	BIC	SABIC
Model 1	0.92	0.91	0.05	0.05	108814.74	109712.64	109217.00
Model 2	–	–	–	–	111208.79	111882.21	111510.48
Model 3	–	–	–	–	111179.82	111887.78	111496.98
	Δ−2 LL (*df*_diff_) = 30.98 (6)^*^						

–, Not applicable. Permanent, *n* = 1821; fixed-term, *n* = 514. Δ−2 LL = loglikelihood difference test; *df*, degrees of freedom.

^*^ *P* < 0.05.

### Main and interaction effects

Table 3 presents findings from the latent moderation: covariates, main effects, and interaction effects by contract type. Covariates age, gender, and civil status accounted for some variance in the health-related outcomes, while children did not.

**Table 3.** Main and interaction effects of predictor variables and covariates on health-related outcomes by contract status with standardized coefficients for all variables as well as confidence intervals for psychosocial working conditions.

Predictor	Permanent contract *β* (Standard error) [95% CI]			Fixed-term contract *β* (Standard error) [95% CI]		
	Health-related outcomes					
	BO	SRH	WFC	BO	SRH	WFC
Age	−0.19*** (*0.03*)	−0.05* (*0.03*)	0.03 (*0.03*)	−0.12** (*0.05*)	0.03 (*0.04*)	0.01 (*0.04*)
Gender	−0.12* (*0.05*)	−0.03 (*0.05*)	−0.10* (*0.04*)	−0.11 (*0.10*)	−0.19* (*0.08*)	−0.07 (*0.09*)
Civil status	−0.21** (*0.07*)	−0.24*** (*0.07*)	−0.01 (*0.06*)	−0.28* (*0.13*)	−0.42*** (*0.12*)	−0.36** (*0.12*)
Children	0.03 (*0.06*)	−0.01 (*0.06*)	0.06 (*0.06*)	0.02 (*0.10*)	0.14 (*0.09*)	0.12 (*0.09*)
Effort	0.43*** (*0.05*) [0.329, 0.525]	0.26*** (*0.04*) [0.177, 0.333]	0.71*** (*0.03*) [0.640, 0.773]	0.34*** (*0.06*) [0.222, 0.461]	0.18** (*0.05*) [0.076, 0.275]	0.63*** (*0.04*) [0.546, 0.707]
Reward	−0.41*** (*0.04*) [−0.487, −0.337]	−0.25*** (*0.03*) [−0.309, −0.189]	−0.05 (*0.04*) [−0.121, 0.016]	−0.43*** ( *0.06*) [−0.550, −0.310]	−0.30*** (*0.05*) [−0.398, −0.201]	−0.07 (*0.06*) [−0.182, 0.048]
ER interaction	−0.15*** (*0.03*) [−0.220, −0.089]	−0.11*** (*0.03*) [−0.159, −0.050]	−0.06* (*0.03*) [−0.111, −0.014]	−0.08 (*0.06*) [−0.197, 0.035]	−0.04 (*0.05*) [−0.150, 0.064]	−0.03 (*0.05*) [−0.117, 0.066]

Permanent, *n* = 1821; fixed-term, *n* = 514.

CI, confidence interval; BO, burnout; SRH, self-rated health; WFC, work-family conflict; ER, effort-reward.

^***^ *P* < 0.001

^**^ *P* < 0.01

^*^ *P* < 0.05.

Statistically significant main effects were found for effort and all health-related outcomes, supporting hypotheses 1a–1c. Regarding reward, hypotheses 2a–c, significant main effects were found for BO (2a) and SRH (2b). However, reward did not significantly contribute to variance in WFC, thus not confirming hypothesis 2c. Hypotheses 3a–c were partially confirmed: the interaction of effort and reward significantly predicted BO, SRH, and WFC among permanent contract employees. Thus, reward moderated the relationship between effort and health-related outcomes, yet only among those with permanent contracts. For the fixed-term employees, the interaction effects were not statistically significant. Findings from the exploratory research question (concerning how the relationships of effort, reward, and their interaction play out among permanent and fixed-term faculty) revealed similar patterns of main effects, while patterns of interaction effects were different. However, main and interaction effects did not differ significantly between permanent and fixed-term faculty.

**Figure 1.**
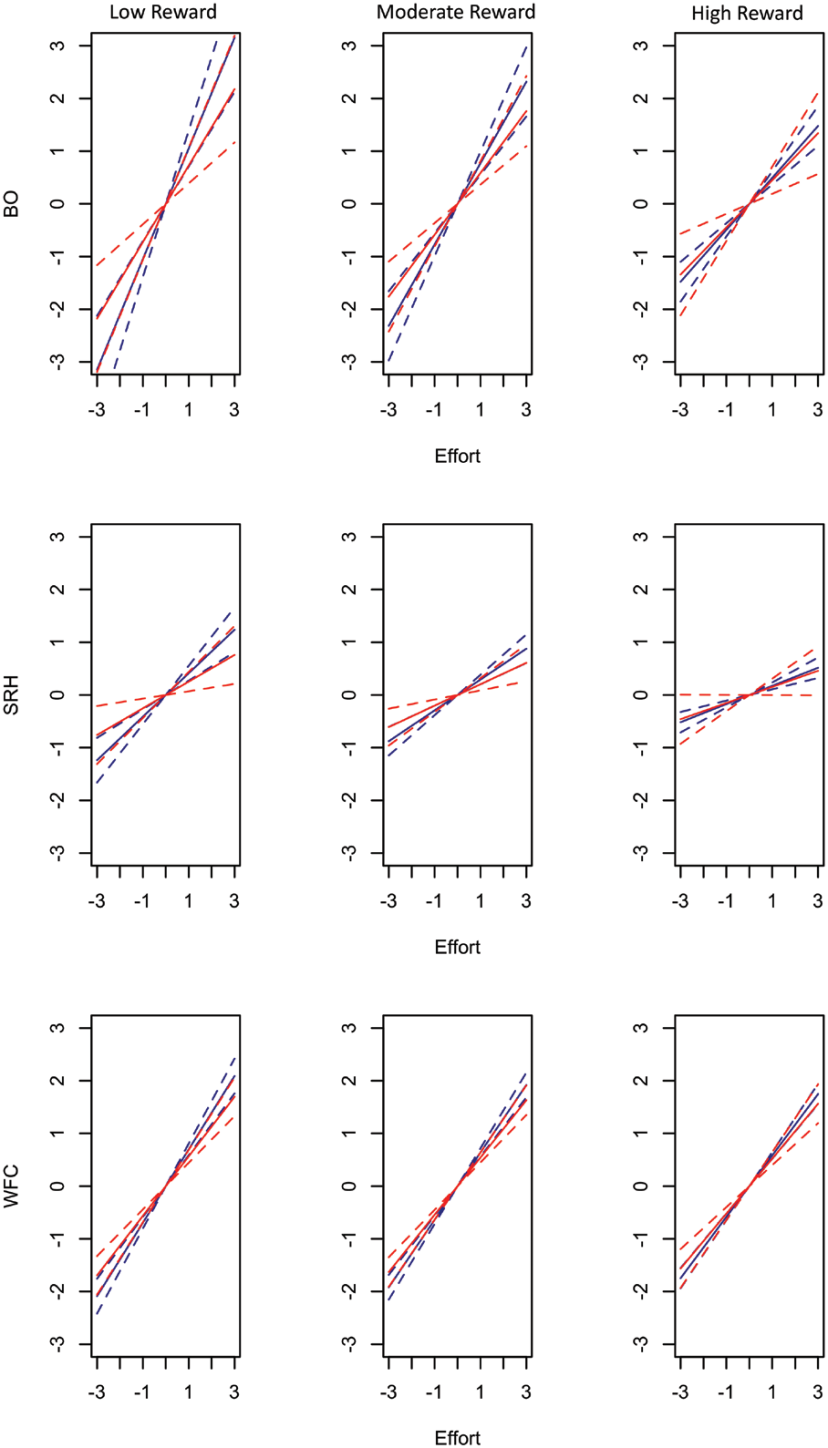
The effect of effort on burnout (BO; top row), self-rated health (SRH; middle row), and work-family conflict (WFC; bottom row) for −1 (left), 0 (center), and +1 (right) standard deviations of reward among employees with permanent contracts (solid blue lines) and fixed-term contracts (solid red lines). The dashed lines represent confidence intervals.

Simple slopes tests revealed that the relationship between effort and burnout for low reward (*b* = 1.05, SE = 0.17, *P* < 0.001) was positive and stronger than for medium (*b* = 0.77, SE = 0.11, *P* < 0.001) and high reward (*b* = 0.49, SE = 0.06, *P* < 0.001) among those with permanent contracts (see Fig. 1). Similar results were found for fixed-term faculty. The relationship between effort and burnout was positive and stronger for low levels of reward (*b* = 0.73, SE = 0.17, *P* < 0.001) than for medium (*b* = 0.59, SE = 0.11, *P* < 0.001) and high levels (*b* = 0.45, SE = 0.13, *P* < 0.01). As for SRH, the relationship between effort and SRH at low reward (*b* = 0.41, SE = 0.07, *P* < 0.001) was positive and stronger than for medium (*b* = 0.30, SE = 0.05, *P* < 0.001) and high reward (*b* = 0.17, SE = 0.03, *P* < 0.001) among permanent faculty. Among fixed-term faculty the relationship between effort and SRH for low reward (*b* = 0.25, SE = 0.09, *P* < 0.01) was positive and stronger than for medium reward (*b* = 0.20, SE = 0.06, *P* < 0.01). Statistically, there were no differences at medium and high levels of reward for the effects of effort on self-rated health (*b* = 0.15, SE = 0.08, *P* = 0.05). Tests of simple slopes for effort on WFC at low reward (*b* = 0.70, SE = 0.06, *P* < 0.001) were positive and stronger than for medium (*b* = 0.64, SE = 0.04, *P* < 0.001) and high (*b* = 0.58, SE = 0.03, *P* < 0.001) reward among permanent employees. For those with fixed-term contracts, simple slopes tests revealed that the relationship between effort and WFC was positive and stronger for low levels of reward (*b* = 0.57, SE = 0.06, *P* < 0.001) than for medium (*b* = 0.54, SE = 0.05, *P* < 0.001) and high (*b* = 0.52, SE = 0.06, *P* < 0.001) levels of reward.

## Discussion

This cross-sectional study investigated the psychosocial working conditions in relation to health-related outcomes, including BO, SRH, and WFC, among faculty in Sweden. Additionally, we explored these relationships for permanent and fixed-term contract employees, respectively, to clarify how contract type influences the relationship between effort, reward, and their interaction, and different occupationally relevant health-related outcomes among faculty. Overall, effort was positively related to BO, SRH, and WFC, while reward was negatively associated with BO and SRH, but not with WFC. Reward buffered the relationship between effort and BO, SRH, and WFC, yet looking at the two contract types, this only seemed to be the case among those with permanent contracts. No significant interaction effects emerged among those with fixed-term contracts. These exploratory findings may suggest that permanent contract faculty may be more affected by the presence or lack of reward than their fixed-term colleagues. Prior studies on working conditions and health-related outcomes among academics provide an empirical foundation for this research (Zábrodská *et al.*, 2018; Kinman, 2019), but this study is one of the first to investigate the psychosocial work environment in association with health-related outcomes among permanent and fixed-term faculty in Sweden. Importantly, we add to the current empirical understanding of effort, reward, and WFC.

Following previous studies (Niedhammer *et al.*, 2004; Gorgievski *et al.*, 2019), effort and reward were significantly associated with BO and SRH. Effort was associated with higher BO and poorer SRH, while reward was related to lower BO and improved SRH. Testing the relationships between effort, reward, and WFC, a significant relationship emerged between effort and WFC, but not for reward, corroborating previous findings (Gorgievski *et al.*, 2019). This may be attributed to the time-based conflict mechanism where time-related demands, including overtime, contribute to WFC due to absence or diminished availability at home. This mechanism may also explain why reward did not significantly predict WFC: the conceptualization of reward and its components may not counterbalance overtime, or absence from family responsibilities. Instead, compensation including overtime pay or compensatory time may be more effective against WFC than rewards including esteem, security or promotion.

Besides investigating main effects, we tested the interaction between effort and reward to explain BO, SRH, and WFC. Among those with permanent contracts, the interaction was significantly associated with BO, SRH, and WFC over and above individual effects of effort and reward, which aligns with theoretical assumptions of the ERI model. Concurrently, the results for fixed-term contract employees do not seem to align with this assumption. This could certainly be explained by a lack of statistical power considering the smaller sample within the fixed-term group. Yet, perhaps for those with fixed-term contracts, low reward may not significantly worsen the effects of effort on the health-related outcomes, and high reward may not alleviate any such effects. One viable explanation for this relates to the situation in academia where permanent contracts tend to be held by those with the most academic merits (e.g. professors). Many fixed-term contracts, especially in Sweden, provide recent PhDs opportunities to acquire merits, increasing their employability and chances of securing a permanent contract in academia. While the reward system including esteem, security, and promotion may benefit fixed-term employee outcomes, perhaps the ultimate reward, that is a permanent contract, is not explicitly accounted for. This might explain why the reward operationalization did not moderate the relationship between effort and health-related outcomes among those with fixed-term contracts.

Examining our findings from a theoretical perspective (Siegrist, 1996), it should be acknowledged that individuals may in fact knowingly accept conditions involving effort/reward-imbalance with the expectation that a period of imbalance will eventually lead to the desired career or position. During such a period, high effort without compensatory reward may be tolerated and have less detrimental effects. This could perhaps provide some explanation as to why the interaction between effort and reward did not contribute to adverse outcomes for those with fixed-term employment, whereas it did among those with permanent employment.

### Limitations and future directions

Regarding limitations, the unequal sample-sizes between permanent and fixed-term faculty hinders comparisons by contract type. Still, these sample sizes do reflect the current circumstances in academia. Moreover, the cross-sectional design allows no conclusions about causality, and importantly, any long-term effects remain unknown. Also, relying only on self-reports involves a risk of common method bias (Podsakoff *et al.*, 2003), yet self-reports are necessary for evaluating subjective experiences. Furthermore, the union members studied do not fully represent all scientific disciplines (under-representation of medical sciences). Finally, the response rate may raise concerns regarding willingness to participate and overall representation. Despite an overall adequate sample size, future studies should aim for increased representativity, especially among fixed-term faculty, and longitudinal designs.

When interpreting statistically significant main and interaction effects, the present findings should be considered in relation to the chosen model, Model 3. While the measurement model and the baseline model (Model 1) met acceptable fit, the fit indices together with factor structure and reliabilities revealed weaknesses regarding the measurement scales. Moreover, without traditional fit indices for Models 2 and 3, it is impossible to ascertain the precision of the final model and whether it met adequate fit cut-offs. However, various relative fit indices (i.e. AIC, BIC, and SABIC), the log likelihood test, and theoretical reasoning resulted in selecting the LMS model as the best approximation. Our findings are consistent with this.

Investigating contract type requires addressing the composition of the fixed-term group. Collapsing those without permanent contracts into one category, *fixed-term*, impedes possibilities to explore nuanced differences between various non-permanent contracts. Importantly, these contract types require closer examination. Still, most in this category held contracts entitled as *fixed-term*. Thus, future research should target faculty with various temporary contract types to understand their circumstances.

### Practical implications

The present findings have practical implications for employees and higher education institutions. The moderating effect of reward among academics with permanent employment and the beneficial direct effects of reward for fixed-term employee health may suggest that higher education institutions should take active measures to bolster various reward systems. Specific initiatives may, for instance, promote collegial recognition and esteem while preventing stress and inspiring others. Addressing reward components of job security and job promotion may involve greater challenges as these depend on employment legislation. However, wherever possible, openness and transparency around promotion opportunities can signal employer readiness and willingness to recognize faculty.

## Conclusions

This study aimed to examine the psychosocial work environment and health-related outcomes among academic employees in Sweden, with an interest in exploring how these relationships play out for permanent and fixed-term employees. Our findings suggest that overall, ERI is relevant in the academic context. However, it is possible that its applicability may depend on contractual circumstances. Thus, this research makes a theoretical contribution regarding the efficacy of ERI across different contractual statuses, in addition to its usage in relation to WFC. Empirically, this study extends current knowledge about working conditions in higher education and how these are associated with health-related outcomes. This is imperative to national and international efforts to ensure sustainable working conditions.

## Data Availability

This research data set includes sensitive information, which if made public, would compromise the integrity of the participants. For questions regarding the research data, please contact Petra Lindfors.
