# Correction to: How do Effort, Reward, and Their Combined Effects Predict Burnout, Self-rated Health, and Work-family Conflict Among Permanent and Fixed-term Faculty?

**DOI:** 10.1093/annweh/wxad038

**Published:** 2023-07-08

This is a correction to: Anna Sofia Tanimoto and others, How do Effort, Reward, and Their Combined Effects Predict Burnout, Self-rated Health, and Work-family Conflict Among Permanent and Fixed-term Faculty?, *Annals of Work Exposures and Health*, Volume 67, Issue 4, May 2023, Pages 462–472, https://doi.org/10.1093/annweh/wxac094

In the originally published version of this manuscript, there were errors in the parameter estimates reported in Table 3. Table 3 should read:

**Table 3.** Main and interaction effects of predictor variables and covariates on health-related outcomes by contract status with standardized coefficients for all variables as well as confidence intervals for psychosocial working conditions.

Predictor	Permanent contract *β* (Standard error) [95% CI]			Fixed-term contract *β* (Standard error) [95% CI]		
	Health-related outcomes					
	BO	SRH	WFC	BO	SRH	WFC
Age	−0.19*** (*0.03*)	−0.05* (*0.03*)	0.03 (*0.03*)	−0.12** (*0.05*)	0.03 (*0.04*)	0.01 (*0.04*)
Gender	−0.12* (*0.05*)	−0.03 (*0.05*)	−0.10* (*0.04*)	−0.11 (*0.10*)	−0.19* (*0.08*)	−0.07 (*0.09*)
Civil status	−0.21** (*0.07*)	−0.24*** (*0.07*)	−0.01 (*0.06*)	−0.28* (*0.13*)	−0.42*** (*0.12*)	−0.36** (*0.12*)
Children	0.03 (*0.06*)	−0.01 (*0.06*)	0.06 (*0.06*)	0.02 (*0.10*)	0.14 (*0.09*)	0.12 (*0.09*)
Effort	0.43*** (*0.05*) [0.329, 0.525]	0.26*** (*0.04*) [0.177, 0.333]	0.71*** (*0.03*) [0.640, 0.773]	0.34*** (*0.06*) [0.222, 0.461]	0.18** (*0.05*) [0.076, 0.275]	0.63*** (*0.04*) [0.546, 0.707]
Reward	−0.41*** (*0.04*) [−0.487, −0.337]	−0.25*** (*0.03*) [−0.309, −0.189]	−0.05 (*0.04*) [−0.121, 0.016]	−0.43*** (*0.06*) [−0.550, −0.310]	−0.30*** (*0.05*) [−0.398, −0.201]	−0.07 (*0.06*) [−0.182, 0.048]
ER interaction	−0.15*** (*0.03*) [−0.220, −0.089]	−0.11*** (*0.03*) [−0.159, −0.050]	−0.06* (*0.03*) [−0.111, −0.014]	−0.08 (*0.06*) [−0.197, 0.035]	−0.04 (*0.05*) [−0.150, 0.064]	−0.03 (*0.05*) [−0.117, 0.066]

Permanent, *n* = 1821; fixed-term, *n* = 514.

CI, confidence interval; BO, burnout; SRH, self-rated health; WFC, work-family conflict; ER, effort-reward.

***P < 0.001, **P < 0.01, *P < 0.05.

instead of:

**Table 3.** Main and interaction effects of predictor variables and covariates on health-related outcomes by contract status with standardized coefficients for all variables as well as confidence intervals for psychosocial working conditions.

Predictor	Permanent contract *β* (*Standard error*) [95% CI]			Fixed-term contract *β* (*Standard error*) [95% CI]		
	Health-related outcomes					
	BO	SRH	WFC	BO	SRH	WFC
Age	−0.19*** *(0.03)*	−0.05* *(0.03)*	0.03 *(0.03)*	−0.12** *(0.05)*	0.03 *(0.04)*	0.01 *(0.04)*
Gender	−0.06* *(0.02)*	−0.01 *(0.02)*	−0.05* *(0.02)*	−0.05 *(0.05)*	−0.09* *(0.04)*	−0.03 *(0.04)*
Civil status	−0.08** *(0.02)*	−0.09*** *(0.03)*	−0.01 *(0.02)*	−0.10* *(0.05)*	−0.16** *(0.05)*	−0.14** *(0.04)*
Children	0.01 *(0.03)*	−0.01 *(0.03)*	0.03 *(0.03)*	0.01 *(0.05)*	0.07 *(0.04)*	0.06 *(0.05)*
Effort	0.77*** *(0.11)* [0.552, 0.990]	0.29*** *(0.05)* [0.202, 0.383]	0.64*** *(0.04)* [0.561, 0.718]	0.59*** *(0.11)* [0.365, 0.808]	0.20** *(0.06)* [0.087, 0.320]	0.54*** *(0.05)* [0.450, 0.637]
Reward	−0.58*** *(0.06)* [−0.701, −0.459	−0.22*** *(0.03)* [−0.278, −0.168]	−0.04 *(0.03)* [−0.085, −0.011]	−0.58*** *(0.10)* [−0.764, −0.389]	−0.27*** *(0.05)* [−0.366, −0.176]	−0.05 *(0.04)* [−0.123, 0.033]
ER interaction	−0.28*** *(0.07)* [−0.414, −0.144]	−0.12*** *(0.03)* [−0.183, −0.057]	−0.06* *(0.02)* [−0.101, −0.012]	−0.14 *(0.10)* [−0.342, −0.063]	−0.05 *(0.06)* [−0.174, −0.074]	−0.02 *(0.04)* [−0.101, 0.057]

Notes: Permanent, *n* = 1821; fixed-term, *n* = 514. Abbreviations: CI = confidence interval, BO = burnout, SRH = self-rated health, WFC = work-family conflict, ER = effort-reward.

***p < 0.001, **p < 0.01, *p < 0.05.

The errors have been corrected in the article.

